# Overexpression of SMPX in Adult Skeletal Muscle Does not Change Skeletal Muscle Fiber Type or Size

**DOI:** 10.1371/journal.pone.0099232

**Published:** 2014-06-17

**Authors:** Einar Eftestøl, Tine Norman Alver, Kristian Gundersen, Jo C. Bruusgaard

**Affiliations:** 1 Department of Biosciences, University of Oslo, Oslo, Norway; 2 Atlantis Medical University College, Oslo, Norway; University of Sydney, Australia

## Abstract

Mechanical factors such as stretch are thought to be important in the regulation of muscle phenotype. Small muscle protein X-linked (SMPX) is upregulated by stretch in skeletal muscle and has been suggested to serve both as a transcription factor and a mechanosensor, possibly giving rise to changes in both fiber size and fiber type. We have used *in vivo* confocal imaging to study the subcellular localization of SMPX in skeletal muscle fibers of adult rats using a SMPX-EGFP fusion protein. The fusion protein was localized predominantly in repetitive double stripes flanking the Z-disc, and was excluded from all nuclei. This localization would be consistent with SMPX being a mechanoreceptor, but not with SMPX playing a role as a transcription factor. *In vivo* overexpression of ectopic SMPX in skeletal muscle of adult mice gave no significant changes in fiber type distribution or cross sectional area, thus a role of SMPX in regulating muscle phenotype remains unclear.

## Introduction

Skeletal muscle displays a remarkable ability to adapt to variable conditions such as electrical activity, hormones and mechanical load. Nerve evoked electrical activity is perhaps the most important stimulus for changing muscle phenotype [1], [2], [3], and the effect of hormones, particularly anabolic steroids, is well known to cause dramatic changes in skeletal muscle tissue [4], [5], [6], [7]. How muscle cells sense and respond to changes in mechanical load has, however, not been investigated to the same extent as the factors mentioned above, and numerous candidate proteins have been suggested as mechanosensors both in cardiac and skeletal muscle [8]. The most likely candidate structure for mechanotransduction in muscle cells are the costameres, demonstrated to be important for lateral force transmission during muscle contraction [9]. These are cytoskeletal protein complexes arranged so that they flank the Z-line and overlie the I-band of sarcomeres and anchor the sarcomeres to the extracellular matrix [10].

One of the candidate proteins for mechanotransduction is Small Muscle Protein X-linked (SMPX), a 9 kD protein predominantly expressed in cardiac and skeletal muscle. It is highly conserved in mammals and is upregulated in skeletal muscle during passive stretch [11], [12]. SMPX is located to the costameres, but has not been verified to play a definite role as a mechanosensor in muscle. However, it has been suggested that SMPX might increase the adhesive function of integrins [13], suggesting a role as an indirect mechanotransductor. Additionally, SMPX is localized to the costameres, both *in vivo* and in C2C12 cells [14], and it co-precipitates with vinculin, a major constituent of the costameres [10], [13].

From a phenotypic perspective, SMPX has been thought to be a candidate gene in regulating muscle size. Schindeler *et al.* (2005) found that SMPX may participate in the regulation of cytoskeletal dynamics through the Rac1/p38 pathway, a pathway involved in mechanosensing in other tissues [15]. Additionally, differentiating C2C12 myoblasts overexpressing SMPX increase their susceptibility to fuse, forming large “myosacs” in an IGF-1 dependent manner [14]. This further indicates a role for SMPX in the regulation of fiber size.

SMPX has also been suggested to regulate another key phenotype in skeletal muscle: Putative binding sites for several muscle-specific transcription factors has been found in the promoter region located immediately 5′ to the *Smpx* gene, including MyoD and MEF-2 [11], [12], [14], indicating that SMPX could be regulated by one or both of these myogenic factors. Overexpression of SMPX in cultured C2C12 muscle cells leads to activation of the transcription factors MEF2 and NFAT in an IGF-1 dependent manner. Both MEF2 and NFAT are key players in muscle differentiation [16], [17] and are implicated in regulating gene expression associated with fiber type changes [2].

NFAT also regulates several genes involved in the determination of muscle phenotype [18], [19] and is predominantly active in slow fibers [18], [19]. SMPX itself is enriched in slow fibers [14], which led us to the hypothesis that increased levels of SMPX in adult skeletal muscle could induce a shift towards a “slow” muscle phenotype. Also, SMPX might play a role in inducing hypertrophy in adult skeletal muscle by increasing the cells' sensitivity to mechanical strain.

Here, we describe the intracellular localization of a SMPX-EGFP fusion protein in adult murine muscle fibers in sedentary and functionally overloaded animals. We also show that overexpression of the untagged protein *in vivo* does not change the phenotype of the muscle cells, with regard to size and MyHC fiber type composition.

## Methods

### Plasmids

**Figure 1:**
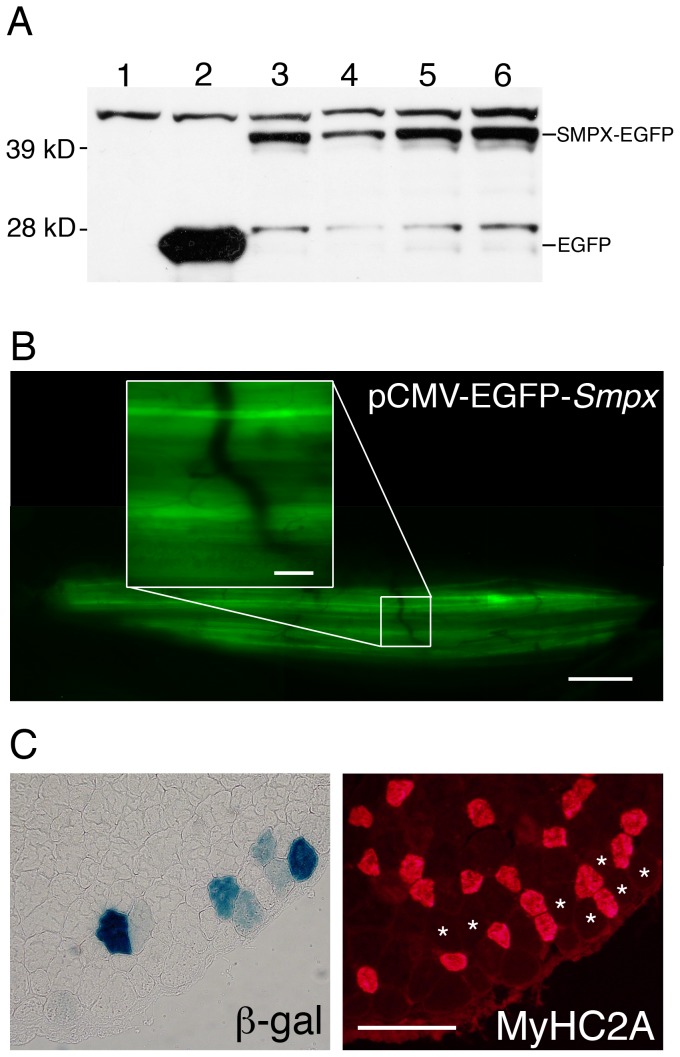
Expression of SMPX plasmids, in vitro and in vivo. A) Western blot of SMPX-EGFP expression in HEK-293 cells stained with antibodies against EGFP. Lane 1: Negative control. Lane 2: EGFP only. Lane 3-6: SMPX-EGFP. B) *In vivo* fluorescence image of EDL muscle expressing pCMS-EGFP-*Smpx*. Scalebar is 500 microns. Inset: High magnification of single fibers. Scalebar is 50 microns. C) β-gal (left) and myosin heavy chain type 2A (right) stain on neighboring cross-sections from the same muscle as in B). β-gal expressing fibers marked with asterisks. Scale bar is 50 microns.

In order to study the subcellular localization of SMPX, a fusion protein expressing SMPX in frame with EGFP (pEGFP-N1-*Smpx*) was made. *Smpx* was inserted at the N-terminal end of EGFP in the plasmid pEGFP-N1 (Clontech). We verified the expression of the SMPX-EGFP fusion protein by western blotting of transfected HEK-293 cells, with a band at the predicted size of approximately 40 kD, using a mouse antibody against GFP (Invitrogen). The negative control and EGFP only control showed no band at the correct size (Figure 1A). To induce *in vivo* overexpression of SMPX in adult muscle fibers, EGFP and *Smpx* driven by separate CMV promoters on the same plasmid (pCMS-EGFP-*Smpx)* were used. The contralateral leg was transfected with pCMS-EGFP (Clontech), serving as a sham control. For the fiber type and cross sectional area analysis a reporter plasmid (pAP-lacZ, gift from N. Gautam) expressing β-galactosidase (β-gal) was used to detect transfected fibers, since EGFP is difficult to detect on cryosections. β-gal was driven by the RSV promoter, while the SMPX-EGFP fusion protein, SMPX and EGFP were all driven by the CMV promoter.

### Cell culture

HEK-293 cells (ATCC: CRL-1573) were grown in DMEM (GIBCO) containing 10% fetal calf serum (Bio Whittaker) and 100 U/ml penicillin and streptomycin. C2C12 cells (ATCC: CRL-1772) were grown as the HEK-293 cells, but with 20% fetal calf serum to maintain the cells in an undifferentiated myoblast stage. All cells were grown with a set temperature of 37°C, a CO2 level of 5% and humidity of 100%.

All transfections were done using FuGENE 6 (Roche) according to manufacturers instructions. As a sham control, one well was always transfected with the pCMS-EGFP-N1 plasmid without *Smpx* inserted. Transfection without any plasmid was used as a negative control (data not shown). All cell culture images shown were obtained 1 day after transfection, with Hoechst 33342 added to the media to visualize nuclei.

### Protein analysis

Transfected HEK293 cells were washed twice with ice cold PBS and lysed with lysis buffer A: (50 mM tris acetate, 0.27 M sucrose, 1 mM EDTA, 1 mM EGTA, 1 mM sodium orthovanadate, 10 mM α-glycerophosphate, 50 mM sodium fluoride, 5 mM sodium pyrophosphate, 1% (w/v) Triton X100, 0.2 mM PMSF, 1 mM benzamidine and 0.1% (v/v) β-mercaptoethanol). The lysates were then centrifuged at 4°C and 13000G for 20 minutes and the supernatant saved. Protein concentration was then measured at 595 nm using a Bradford assay on Victor 2 (Wallac). 30 µg of protein was run on NuPAGE Novex Bis-Tris 4–12% Gels (Invitrogen), followed by Western blotting (NuPAGE Western Transfer Protocol, Invitrogen). Blots were developed using the ECL Western Blotting Detection kit (Amersham) according to the manufacturer's instructions. A mouse anti-GFP antibody (Invitrogen) was used to detect SMPX-EGFP and EGFP (Figure 1A). Expression of pCMS-EGFP-*Smpx* was verified with northern blotting on HEK-293 cells (data not shown)

### Animal experiments

The surgical procedures were performed either with male Wistar rats weighing 300–400 g for the localization experiments or with NMRI mice weighing 20–30 g for the isolated whole muscle localization and overexpression experiments. All animals were under deep anaesthesia induced either by Forene® gas (Isoflurane, Abbott) or by intraperitoneal injections of 5 µl g^−1^ Equithesin (Veso Pharmacy, Norwegian School of Veterinary Science, Norway). In order to overload the *extensor digitorum longus* muscle (EDL), approximately two-thirds of the distal end of the *tibialis anterior* muscle was excised. The animals were euthanized by cervical dislocation while anaesthetised. The animal procedures were reviewed and approved by the Norwegian Animal Research Authority and were conducted in accordance with the Norwegian Animal Welfare Act of December 20^th^, 1974, no. 37, chapter VI, sections 20–22, and the Regulation of Animal Experimentation of January 15^th^, 1996.

### *In vivo* protein expression

*In vivo* electroporation with DNA was performed essentially as described previously [20], with some modifications. The muscle was surgically exposed, and 10 µl of DNA solution (1 mg/ml in 0.9% NaCl) was injected into the belly of the muscle. An electrical field was then applied to the muscle using a pulse-generator (Pulsar 6bp-a/s, FHC inc., USA), using five trains of 1000 symmetrical bipolar square pulses lasting 200 µs in each polarity direction, with total amplitude of 20 V for mice and 100 V for rats.

### Immunohistochemistry

#### Sections

Muscles were frozen by submersion in melting isopentane in a slightly stretched condition, and histological analyses of β -gal, MyHC fiber type composition and CSA were determined 14 days after electroporation on 10 µm transverse serial sections, essentially as described previously [21]. MyHC fiber type was determined using the monoclonal antibodies BA-D5 (I), SC-71 (IIa), BF-35 (non-IIx) and BF-F3 (IIb) (Schiaffino et al. 1989) with the secondary antibody Anti-mouse IgG-FITC (F-9137, Sigma) for MyHC I, IIa and non-IIx, and Anti-mouse IgM-Cy3 (J115-165-020, Jackson Immuno Research) for MyHC IIb.

#### Single fibers

Rat EDL were electroporated with pEGFP-N1-*SMPX* or the sham plasmid pEGFP-N1. 24-36 hours after transfection muscles were isolated and placed in ringer solution with an electrolyte composition of (in mM) 2 CaCl_2_ 110 NaCl, 5 KCl, 1 MgCl, 25 NaHCO, 11 glucose, 0.3 glutamic acid, 0.4 glutamine, 5 HEPES bubbled with 5% CO_2_/95% O_2_ and placed under a Confocal microscope for *in situ* imaging of SMPX-EGFP. The muscles were immediately after imaging injected with 1% paraformaldehyde and fixed in 4°C over night. Muscles were then washed in PBS and placed under a fluorescence microscope. Small fiber bundles were mechanically free dissected and placed in permeabilization-buffer (50 mM glycine, 0.25% BSA, 0.04% saponin in 0.01 M PBS) for 2.5 hours at room temperature, and then stained with Alexa Fluor 680 Phalloidin (Invitrogen) in staining solution (1% BSA, 0.5% Triton X-100 in 0.01 M PBS) for 1.5 hours at 32°C. Fibers were then washed 3 times 30 minutes in staining solution without any antibody present and mounted on slides with ProlongGold antifade agent with DAPI (Invitrogen, Oregon, USA). Non-fluorescent fibers were used as negative controls (data not shown). (Modified protocol from Averbeck et al., 2007).

### Confocal microscopy

Imaging with a confocal microscope (Olympus BX61WI, Tokyo, Japan) connected to an imaging system (Olympus fluoview FV1000, Olympus, Europe GmBH) was performed either *in situ* or on isolated fibers from the SMPX-EGFP fusion protein experiments. All images were taken with either a 60X water (LUMPlanFI, NA 0.9) (used *in vivo* and *in situ*) or a 100X oil (UPlan Apo, NA 1.35 oil Iris) (used *in vitro*) immersion objective. Images were further processed in Photoshop CS3- or CS5- extended version (Adobe Systems, San Jose, CA, USA).

Mouse EDL muscle were electroporated with pEGFP-N1-SMPX either without or with overloading by synergist ablation of the *tibialis anterior* muscle. After confirming fluorescence *in vivo* (data not shown), muscles were placed in a 4% paraformaldehyde solution with Hoechst 33342 and further imaged with a confocal microscope.

### Statistical analyses

All statistical calculations were done in Prism4 (GraphPad Software Inc., San Diego, CA, USA), with a level of significance set to 5%. The fiber type distribution in sham control and SMPX muscle fibers were compared using a chi- square test. The CSA measurements were compared using a Students t-test.

## Results

### Nuclear localization

**Figure 2:**
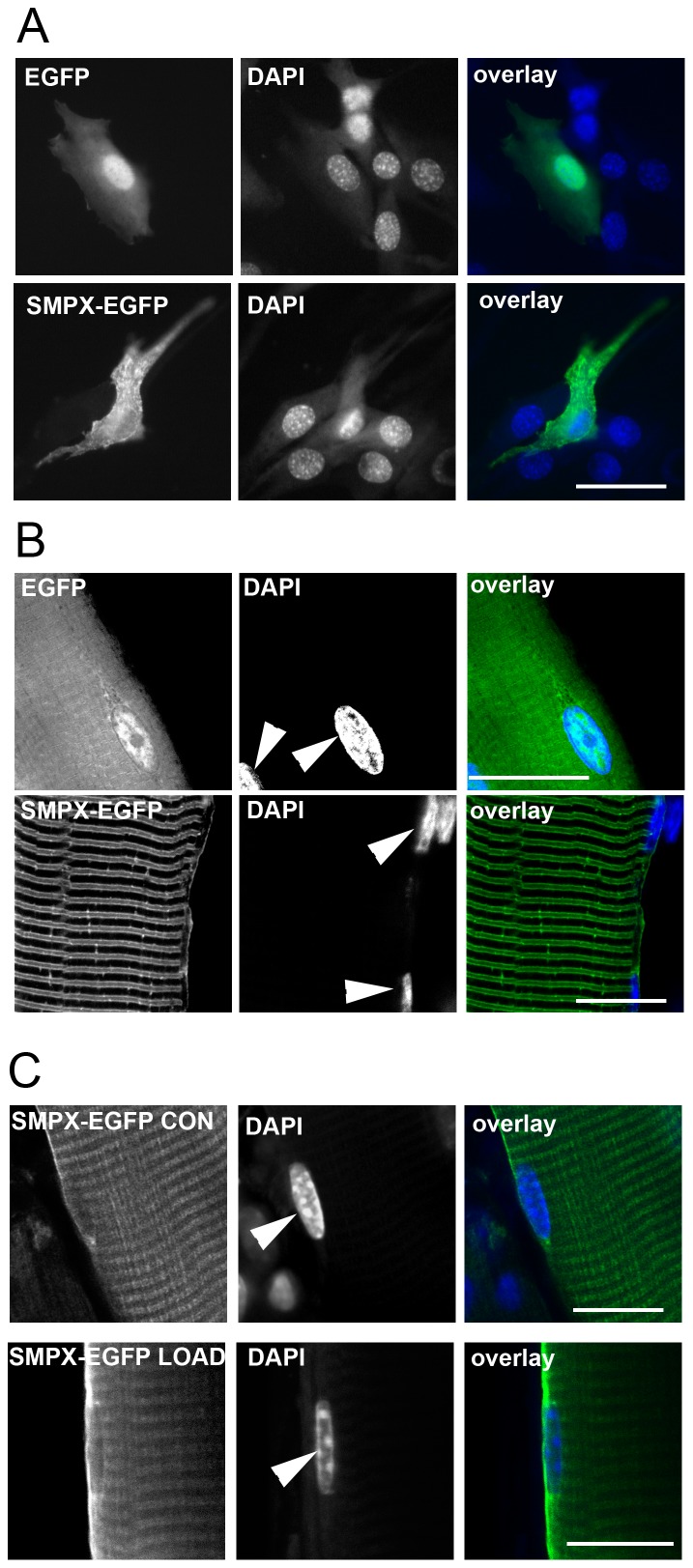
SMPX-EGFP was excluded from all myonuclei. A) C2C12 myoblasts expressing either EGFP or SMPX-EGFP stained with Hoechst 33342 to visualize nuclei. B) *In vitro* confocal image of dissected single rat EDL muscle fibers expressing EGFP or SMPX-EGFP stained with DAPI to visualize nuclei. C) Confocal images of EDL muscle fibers *in situ,* after no treatment (CON) or functional overload for 18 hours (LOAD), expressing SMPX-EGFP stained with Hoechst 33342 to visualize nuclei. Myonuclei are labeled with arrowheads. Scale bar is 10 microns.

Our results show that SMPX-EGFP did not accumulate in the nuclei in cell culture (Figure 2A), levels were below those in the cytosol. In adult skeletal muscle fibers, SMPX-EGFP was not seen above background levels in any investigated nuclei (Figure 2B). In the control experiments with EGFP alone, the signal was localized to the nuclei in much the same intensity as in the cytosol, i.e. no accumulation of protein in the nucleus. The fusion protein was also excluded from the nuclei after 18 hours of overload (2C). As has been described in earlier reports we noticed an increased level of SMPX in the perinuclear area. This was observed in fibers from both normal and overloaded muscles (Figure 2B and 2C) (see also [12], [13]).

### Cytoplasmic localization

SMPX-EGFP had a patchy localization in the cytoplasm of the myoblasts, (Figure 2A). This is in contrast to Palmer *et al.* (2001), who found a different GFP-SMPX fusion protein localized to the leading end of lamellipodia and at focal adhesions.

**Figure 3:**
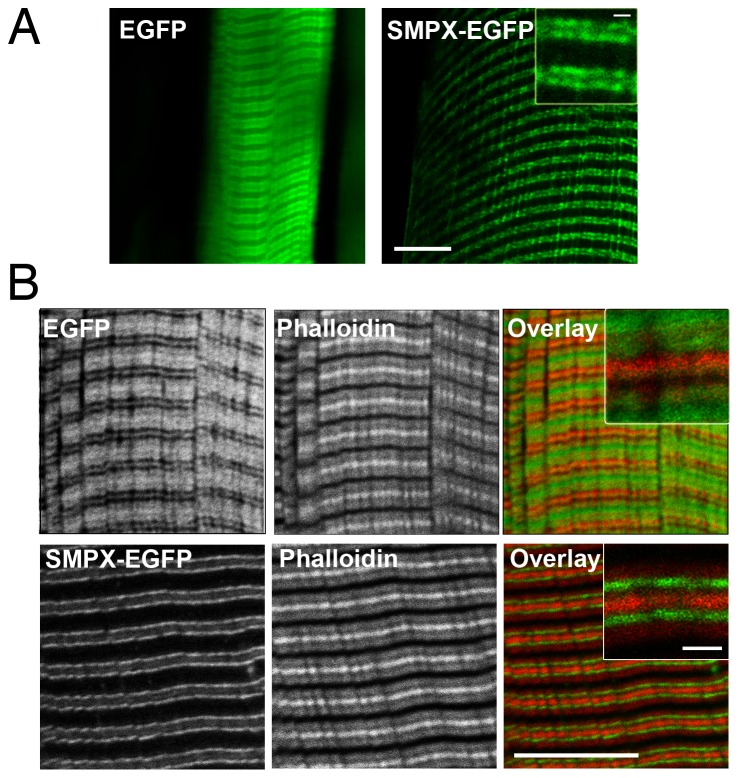
SMPX-EGFP was localized in bands flanking the z-disc. A) *In situ* confocal image of EGFP or SMPX-EGFP expression from an isolated EDL rat muscle in Ringer-solution. Scale bar is 10 microns. B) *In vitro* confocal image of EGFP or SMPX-EGFP stained with Alexa Fluor 680 Phalloidin (red) to visualize actin filaments. Scale bar is 10 microns (inset 1 micron).

Muscles from adult rats transfected with the SMPX-EGFP fusion protein were excised and immediately placed under the microscope for imaging. We found that the protein was forming thin lines appearing as doublets, the pattern being distinctly different from the broad, more diffuse lines seen with EGFP alone (Figure 3A).

To increase the resolution, fibers were fixed, teased, stained with phalloidin and imaged using confocal microscopy. Similarly as in the *in vivo* and *in situ* experiments, SMPX-EGFP appeared to be localized in narrow bands flanking the z-discs (Figure 3B), occasionally with a band overlying the Z-disc itself (data not shown).

### Fiber type and fiber size

**Figure 4:**
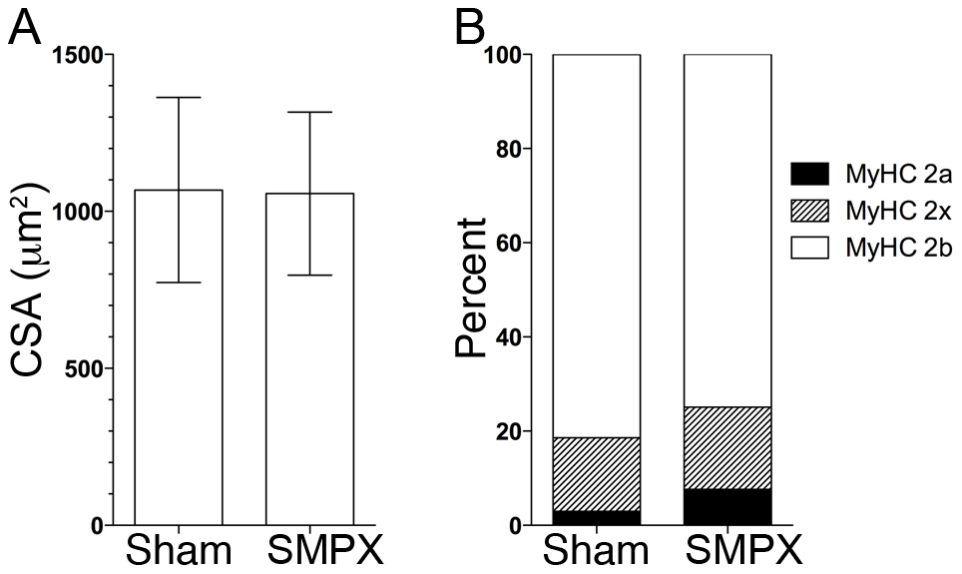
Muscle fiber type and cross sectional area (CSA) in mouse EDL. Single fibers from muscles transfected with either pCMS-EGFP (left leg sham control, only expressing EGFP) or pCMS-EGFP-*Smpx* (expressing SMPX and EGFP). A) CSA with n = 226/254 cells for sham/SMPX. Values are means +/- SD. B) Fiber type composition of the same fibers as in A.

The increased levels of SMPX did not result in a change in cross sectional area (CSA) of the fibers. (Figure 4A). Also, we found no significant effect on fiber type distribution by overexpressing the ectopic SMPX protein (Figure 4B).

## Discussion

It has previously been postulated that SMPX may serve as a transcription factor. In our study though, SMPX-EGFP was not detected above background levels in the nuclei in adult skeletal muscle, and did not show any nuclear accumulation in C2C12 cells compared to cytoplasmic levels. Our findings are in agreement with previous cell culture studies of endogenous and myc-epitope tagged SMPX, that were localized predominantly to the cytoplasm in C2C12 myocytes and myotubes [13], [14]. Also in agreement with our main finding SMPX appears to be excluded from multinuclear muscle cells: Kemp and collaborators found SMPX to be accumulated at varying levels within nuclei of mononuclear C2C12 cells using immunohistochemistry, but absent from all nuclei in fused myotubes [12].

To test whether introducing increased mechanical load could induce nuclear translocation *in vivo*, we subjected the muscle to increased load and stretch by synergist ablation (see methods), a protocol that over time results in substantial hypertrophy [22]. After 18 hours, no visible differences were found between the normal and the overloaded muscles with regard to nuclear accumulation of SMPX. Together with our finding that overexpression of SMPX in the fast EDL muscle had no effect on either fiber cross sectional area or fiber type composition (Figure 4) this speaks against a gene regulatory role for this protein.

We next asked if the subcellular localization of SMPX-EGFP could be consistent with a role as a mechanosensor. In agreement with previous staining of fixed tissue [14] we found the SMPX-EGFP fusion protein to be localized to the level of the costameres that is aligned with the I-bands, flanking each Z-line. This localization might be consistent with SMPX working as a mechanosensor [10].

It remains unclear if SMPX has any major effect on adult muscle phenotype, directly or indirectly. Our findings demonstrate that increased levels of SMPX by overexpression in single adult fibers do not change the fiber type or size. Also, SMPX null-mice do not show any clear phenotypic alterations in skeletal muscle [14]. Interestingly, SPMX might play an important role in another tissue that relies on mechanotransduction, namely the auditory system. Huebner et al. suggest that SMPX plays a critical role in protecting against mechanical stress in cells in the organ of Corti as nonsense mutations in the *Smpx* gene results in hearing loss in humans [23], [24]. The fact that Rac1, a GTPase activated by biomechanichal stress, is a major target for SMPX [13] supports its role as a possible mechanotransducer. Patients from the families in the Huebner study did not show any obvious signs of muscular dysfunction [23], and our results demonstrate that overexpression of the protein does not change fiber type or size. However, it cannot be excluded that decreased levels of the SMPX protein could influence adult skeletal muscle, or that it could serve as a mechanosensor resulting in phenotypic alterations not investigated in this paper.
